# Proceedings of the Wisconsin State Dental Society

**Published:** 1871-10

**Authors:** Chas. C. Chittenden


### Proceedings of Societies.

---

### PROCEEDINGS OF THE WISCONSIN STATE DENTAL SOCIETY, HELD AT LA CROSSE, JULY 11th and 12th, 1871.

The Second Annual Session of the Wisconsin State Dental Society, was held at La Crosse, on July 11th and 12th, 1871.

Society called to order by D. W. Perkins, President.

Roll called by the Secretary.

Present—Drs. D. W. Perkins, J. C. Lukes, C. C Chittenden, E. Palmer, A Holbrook, E. R. E. Carpenter.

Minutes of the previous meeting were read and approved.

The Committee on Membership reported the names of the following dentists as eligible for membership:

Dr. William Decker, of Oshkosh; Dr. R. S. Wells, of Sparta; D. W. Vanburgh, of La Crosse; E. G. Hazelton, of Kenosha; F. A. Williamson, of Red Wing, Minn.; S. W. Gilman, of Beaver Dam, who were severally elected.

The President then made a short address.

On motion of Dr. Palmer, the resident physicians, dentists and members of the press, of La Crosse, were invited to be present and take seats with the society.

The Secretary made his annual report.

The number of dentists signing the Constitution and paying initiation fee, thus becoming active members, was twenty.

Number of honorary members elected, five.

Amount of money paid over to the Treasurer, $100 (one hundred dollars.)

Orders drawn on treasury by vote of society, $26 42.

The Treasurer made his annual report.

The total receipts for the year, previous to the present meeting, $100.

Disbursements by bills paid, $26 42.

Cash advanced to Chairman of Publishing Committee for contingent expenses, $20.

Funds in the hands of the Treasurer, $53 58.

On motion, Drs. Wells, Hazelton and Decker, were appointed to nominate officers for the ensuing year.

The society then adjourned till 2 o'clock P. M.

AFTERNOON SESSION.

Committee on nominations of officers reported the following, who were duly elected:

*President*—Dr. E. Palmer, of La Crosse.

1st. *Vice President*—Dr. A. Holbrook, of Waukesha.

2d. *Vice President*—Dr. J. C. Lukes, of Racine.

*Recording Secretary*—Dr. Chas. C. Chittenden, of Madison.

*Corresponding Secretary*—Dr. D. W. Perkins, of Milwaukee.

*Treasurer*—Dr. A. Solliday, of Watertown.

A committee of three was appointed to wait on the President elect, and escort him to the chair, where he was received by the retiring President, Dr. D. W. Perkins, with appropriate remarks of congratulation.

Dr. Palmer, in responding, said he was duly thankful to the society for the honor conferred upon him; that his heart was set upon the society's success and its object, which was to elevate and bring into a bond of progressive union the dental profession.

The first subject for discussion, Nature of Dental Caries, was taken up.

Dr. W. H. Atkinson, of New York city, being present, was invited to open the subject. He said the subject was so occult and hidden, that it was nearly impossible to make a connected argument without some regularly laid down statements on which to start a discussion.

"*The nature of caries.*" If we really understood the word "nature," the rest would open to us like a barn door. At the word "nature" there rises before us a vision of the whole structure of the tooth in all its various stages and wonderful minuteness of formation. A tooth once properly formed and perfectly constructed, can not possibly decay. There enters into the construction of the tooth, three entirely distinct processes. The enamel may be considered as strictly mineral, and falls properly under the head of chrystalosophy. The dentine is a compound formation of mineral and animal tissue, and may be classed under the heads both of chrystalosophy and cellosophy. The pulp involves the principle of corpusculosophy. If these are all perfectly formed there can be no decay. Decay is the result of a violation of nature's laws during formation. The law of life is action. Health is obedience, decay disobedience of that law. The cause of imperfect formation of the teeth—the planting of the germs of disease, is mainly the result of misdirected parental affection, which not only fails in passively effecting good but actively effects evil, and this has brought into existence the latest upheaval of science, the dental profession.

Dr. Chittenden considered caries entailed upon offspring, and that its development was influenced to a greater or less degree by surrounding influences which might either increase or decrease its celerity and extent.

On motion, the "question and answer" mode of discussion was adopted.

Dr. Wells gave the case of a child four years old in whose mouth every tooth was carious, and asked for the best mode of treatment, with a view to secure a healthy permanent dentition.

Dr. Atkinson thought the condition described was the result of exhaustion of the cells of development. He advised feeding the child upon a confection of prep. chalk, sugar and gum paste. At night make a mortar of prep. chalk about the teeth. In the morning brush with a soft brush the upper teeth downward and the lower teeth upward.

Dr. Hazelton asked: "Will a disease of temporary teeth injure the permanent teeth?"

Dr. Atkinson said not at all, if properly treated. In case of alveolar abscess, after opening and cleansing sac, inject into it elixir of vitriol or pure sulphuric acid. When the necrosed portion of the bone has been sufficiently saturated, wash out the cavity and apply a little tincture of calendula. This treatment will insure a removal of the trouble.

The society then adjourned until 8 o'clock P. M.

EVENING SESSION.

Society called to order by the President.

Discussion of second subject, "Pathological condition of Dental Nerves," occupied the space of about an hour, and was participated in by Drs. Palmer, Wells, Decker, Atkinson, Carpenter and others. The drift of all the arguments was in support of the proposition that dental nerves in almost every stage of disease, where there is vitality in any portion of them could and should be saved alive. The use of oxychloride of zinc was universally advocated as the best covering for exposed pulps.

Dr. Atkinson said inflammation should be treated on general principles systemically. Locally, he formerly used creosote, but now advocated in its place, thymol which he has been using with great success. His mode of treatment was this, after removing caries from cavity, wash the exposed pulp with salted water and calendula, injecting into the tooth with a syringe. Afterwards dress with thymol until the inflammation subsided, then cap with oxychloride of zinc.

Dr. Taylor, of La Crosse, gave his experience in endeavoring to preserve the life of a dental nerve, by the old method, which proved unsuccessful. Stated that with the use of thymol he had been successful in every case he had undertaken.

Dr. Atkinson was elected an honorary member of the society by acclamation.

The President then announced the standing committees for the ensuing year:

*Executive Committee*—Drs. E. W. Foster, R. S. Wells, L. C. Stewart, E. M. Clark, M. B. Johnson, E. G. Hazelton, C. C. Chittenden, C. W. Barnes and Wm. Decker.

*Dental Ethics*—Drs. D. W. Perkins, N. H. Drew and F. A. Williamson.

The President announced that arrangements had been made for some clinical operations to which the time of the morning session would be devoted.

The society adjourned till 8 o'clock A. M., next day.

WEDNESDAY MORNING SESSION.

This session was devoted to clinics. Three operating chairs were brought into requisition.

Dr. E. R. E. Carpenter performed the operation of preparing a large molar crown cavity entirely by means of the Pneumatic engine, and demonstrated beyond a doubt its superiority over any other instrument ever used for that purpose. He filled with Atkinson's foil No. 120, cutting down and polishing the filling with the engine.

Dr. Perkins made two beautiful contour fillings in central incisors.

Dr. Atkinson operated on an exposed pulp, demonstrating his method of treatment for such cases.

Dr. Poor, of Dubuque, Iowa, exhibited a machine for drilling cavities, which when properly developed and perfected will probably be a considerable improvement over the old hand drilling method.

Dr. J. Taft, of Cincinnati, being present, was invited to address the society in the evening.

Adjourned till 2 P. M.

AFTERNOON SESSION.

Society met pursuant to adjournment.

Subject for discussion, "Absorption or Recession of Gums." Discussion was participated in by Drs. Taft, Holbrook, Atkinson and Chittenden.

Dr. Atkinson in cases where there was a deposit of salivary calculus, advocated after removing the deposit, the use of the Pneumatic engine, with porte polisher of wood and prep. chalk, after which he used "dilute elixir of vitriol," applied at the juncture of the tooth and gums, care being taken to prevent its coming in contact with other parts of the mouth, maintaining that the acid would not act chemically on the live dental tissue, but only decompose the remaining particles of lime deposit, impossible to be removed mechanically.

Dr. Holbrook read a paper on atrophy and its causes, for which he received a vote of thanks.

Dr. Taft said the subject was obscure; critical exceptions might be taken to the name given the disease, yet it is appropriate enough for all practical purposes. Atrophy is imperfect organization. There is a great difference in the structure of dentine as seen under the microscope. These pits were never filled by nature. Sometimes the tubuli passed through the dentine and through the enamel, and a microscopic aperture is left in which impurities lodge, and decay is produced, which appears like atrophy but should not be mistaken for it. It is a different disease, but practically requires no different treatment.

We should instruct parents during their children's dentition, to avoid the diseases of childhood, taking good care of the health, feeding the children on proper diet.

Treatment—If the atrophy is superficial, dress down the surface; if deep, or showing tendency to decay, cut out and fill.

Dr. R. F. Wells then read an essay on "Dentistry *vs.* Money," for which he received a vote of thanks.

After discussing this subject at some length, the following resolution was adopted:

*Resolved*, That the society offer a prize of $10 for the best written tract on a subject connected with the care of the teeth, said tract to be written in a style adapted to the popular wants.

A committee of three consisting of Drs. Wells, Decker and Gilman, were appointed to act as judges.

Adjourned till 8 P. M.

EVENING SESSION.

Society met pursuant to adjournment.

President Palmer in the chair.

Chairman of the Publishing Committee presented a bill of M. J. Cantwell & Co., for printing two hundred and fifty copies Constitution, By-laws, etc., $22 50, which was ordered paid.

On motion of Dr. Perkins, a *per capita* tax or assessment of three dollars was voted, and the Secretary instructed to collect it.

On motion of Dr. Holbrook, the Publishing Committee was instructed to secure the publication, in pamphlet form, of the entire proceedings of the society from its organization.

Dr. Taft then delivered an able address on the Standing of the Dental Profession. He said: It is necessary to take an exact reckoning that we may decide thereby what is ahead of us, and how we shall go in the future. What have we? A profession young, but strong, accomplishing even more than it has dared to claim until recently. It has not grown in a hot bed, but has fought its way through many tribulations. Stabbed in the house of its friends, with no assistance from without, yet, to-day, it challenges the admiration of the world. Within ten years over one hundred societies have sprung up, aiding and advancing the profession. Earnest men always attract attention, and if on the right track will succeed.

Another means of our advancement is our *Dental Literature*. This is not now what it might be, because it is not properly supported by the pens and purses of the profession at large.

Another powerful influence for our professional advancement, is our dental colleges. These should demand a higher standard of qualifications for admission and graduation. It is delightful to observe that the profession is growing daily more hungry for knowledge. The more we learn the more we find there is to be learned.

The Chairman of the Executive Committee presented a bill of $15 15 for postage, publishing notices, etc. This was allowed and an order drawn.

On motion, Dr. Taft was declared an honorary member of the society.

Dr. Palmer introduced the following amendment to Constitution:

*Resolved*, That it shall be the duty of the Secretary to strike from the list of active members, the name of any person who has failed or may fail to participate in the meetings of this society, and pay his dues to the same, for two successive years, and the person so expelled shall not be restored again to membership, except by vote of two-thirds of all the members present at any regular meeting.

Laid over according to Constitution.

Dr. Palmer moved to amend the By-laws by inserting the word three in place of five, in section 9.

Laid over till January meeting.

Dr. Chittenden offered the following:

*Resolved*, That this society individually and collectively, owe a debt which we know not how to express or repay to Drs. Taft and Atkinson, for their presence and assistance to us in our deliberations and discussions at this meeting—they having come a great distance to be with us; that we offer them our warmest thanks. Adopted.

Dr. Palmer offered the following, which was adopted:

*Resolved*, That the thanks of the society are hereby tendered to Twilight Lodge of Good Templars, for the use of their hall during the session of the society.

Dr. Wells offered the following:

*Resolved*, That the thanks of the society are hereby tendered to Mr. C. F. Wilkins, for the use of one of his best chairs during the clinical operations. Adopted.

The following gentlemen were appointed delegates to "American Dental Association:" Drs. C. C. Chittenden, R. S. Wells, D. W. Perkins, F. A. Williamson, S. M. Gilman.

The society then adjourned, to meet at Watertown, on the second Tuesday in January, 1872.

CHAS. C. CHITTENDEN, *Sec.*

